# Causes of death of patients with non-valvular atrial fibrillation in Asians

**DOI:** 10.1371/journal.pone.0282455

**Published:** 2023-03-01

**Authors:** Rungroj Krittayaphong, Thanita Boonyapiphat, Suchart Aroonsiriwattana, Pornchai Ngamjanyaporn, Gregory Y. H. Lip

**Affiliations:** 1 Faculty of Medicine Siriraj Hospital, Department of Medicine, Division of Cardiology, Mahidol University, Bangkok, Thailand; 2 Lampang Hospital, Lampang, Thailand; 3 Surat Thani Hospital, Surat Thani, Thailand; 4 Chonburi Hospital, Chonburi, Thailand; 5 Liverpool Centre for Cardiovascular Science at the University of Liverpool, Liverpool John Moores University and the Liverpool Heart & Chest Hospital, Liverpool, United Kingdom; 6 Faculty of Medicine, Department of Clinical Medicine, Aalborg University, Aalborg, Denmark; BSMMU: Bangabandhu Sheikh Mujib Medical University, BANGLADESH

## Abstract

**Objectives:**

The aim of this study was to determine the causes of death among Asian non-valvular atrial fibrillation (AF) patients who were registered in a nationwide AF registry, and to investigate the differences in the causes of death in AF patients compared between those who were taking and not taking oral anticoagulant (OAC).

**Methods:**

The **CO**hort of antithrombotic use and **O**ptimal INR **L**evel in patients with non-valvular **A**trial **F**ibrillation in Thailand (COOL-AF) study enrolled non-valvular AF patients from 27 centers in Thailand during 2014–2017 to create the COOL-AF Thailand registry. Cause of death was classified as cardiovascular (CV) death, non-CV death, or undetermined cause of death. All events were evaluated and verified by an independent adjudication committee.

**Results:**

There was a total of 3,405 patients (mean age: 67.8 years, 41.8% female), and the mean follow-up duration was 31.8±8.7 months. Three hundred and eighty patients (11.2%) died during follow-up. CV death, non-CV death, and undetermined cause accounted for 121 (31.8%), 189 (49.7%), and 70 (18.4%) patients, respectively. Of those with a known cause of death, heart failure (10%), intracranial hemorrhage (ICH; 10%), sudden cardiac death (6.8%), and ischemic stroke (5.8%) were the most often observed causes of death. Concerning non-CV death, infection/sepsis (27.7%), cancer (5.5%), respiratory (5.2%), and major bleeding (4.5%) were the most prevalent causes of death. The use and type of OAC were found to be major determinants of ICH and major bleeding incidence.

**Conclusion:**

Death due to ischemic stroke was responsible for only 4.7% of all deaths in Asian AF patients. Non-CV death, such as infection/sepsis or malignancy, was more far more prevalent than CV death in Asian AF patients. An improved integrated care approach is needed to reduce the prevalence of non-CV death in Asian AF patients.

## Introduction

The prevalence of non-valvular atrial fibrillation (AF) is increasing in older adults [1]. AF is a known cause of many serious complications, such as ischemic stroke from thromboembolism, heart failure, and cardiovascular death, and is associated with increased hospitalizations and healthcare costs [2,3]. Oral anticoagulants (OAC) are routinely prescribed to prevent ischemic stroke in AF patients; however, the use of OACs was reported to be associated with an increased incidence of major bleeding in Asian AF patients [4,5].

Many AF patients have comorbid conditions and adherence to an integrated care management strategy was shown to yield better clinical outcomes, including a reduction in death, stroke, bleeding, and hospitalizations [6]. Awareness of the incidence rate and causes of death of AF patients would be beneficial for focusing and improving an integrated care management strategy to care for AF patients. Healthcare systems vary widely from country to country and some treatments shown to be cost-effective in Western populations may not be cost-effective in limited resource settings [7]. For example, direct oral anticoagulants (DOACs), which have been proven to be at least as effective as warfarin for stroke prevention and cause less intracranial bleeding than warfarin, are still used less than warfarin in many Asian countries [8–10]. Differences in treatment options and patterns of antithrombotic use may lead to differences in the causes of death of AF patients among countries. Improved understanding of these important issues will improve both strategic healthcare planning and patient outcomes.

Accordingly, the aim of this study was to determine the causes of death among Asian non-valvular AF patients who were registered in a nationwide AF registry, and to investigate the differences in the causes of death in AF patients compared between those who were taking and not taking OAC.

## Materials and methods

Patients aged greater than 18 years and diagnosed with non-valvular AF were enrolled from 27 centers in Thailand into the **CO**hort of antithrombotic use and **O**ptimal INR **L**evel in patients with non-valvular **A**trial **F**ibrillation in Thailand (COOL-AF) study/registry during 2014–2017. The exclusion criteria were 1) rheumatic valvular disease, 2) mechanical heart valve, 3) ischemic stroke within 3 months, 4) pregnancy, 5) inability to attend follow-up visits, 6) hematologic disease that increased the risk of bleeding, 7) AF from transient and reversible cause, and 8) refusal to participate. The protocol for this study was approved by the Institutional Review Board of each of the 27 participating sites, and every patient that was enrolled provided written informed consent to participate in the study. The study complied with all of the principles and guidelines set forth in the 1964 Declaration of Helsinki and all of its subsequent amendments.

### Study protocol

After obtaining written informed consent, each patient was interviewed and baseline data were recorded in a case record form. The data from the original case record form was then uploaded into a web-based system. All submitted data were quality checked and verified for completeness by a central data management team. The hard copy of the case record form was sent to the central data management team for double entry and verification of data accuracy. Data-related questions or concerns were addressed to investigators at their respective study. Study monitoring was performed at all 27 study sites to ensure the quality of the data, and to confirm that investigators at each site conducted the study in accordance with both good clinical practice guidelines and the study protocol.

### Data collection

Demographic data, vital signs, characteristics of AF, medical history, laboratory data, investigations data, and prescribed medications were collected at baseline. The same types of data were collected again at the 6-, 12-, 18-, 24-, and 30-month follow-up visits. During the follow-up, adverse event data were also recorded.

### Outcomes

The primary outcome of this study was death and the cause(s) of death. The date of death was recorded and all deaths were evaluated by at least 2 members of an independent adjudication committee. The events adjudication committee developed standard procedures to assess the cause of death. Cases that were difficult to conclusively discern were discussed during adjudication team meetings.

Information specific to any death was reported by local investigators using a standardized case record form in addition to a structured narrative description of the event. The details of the death and the cause of death were then uploaded to the web-based system. When available, supporting documents, such as the death certificate, hospital discharge summary, and other records (e.g., radiology, laboratory, and pathological data), were also uploaded into the web-based system.

The cause of death was classified as cardiovascular (CV) death, non-CV death, or undetermined cause of death. CV death was defined as death due to heart failure, ischemic stroke, intracranial hemorrhage (ICH), acute myocardial infarction (AMI), sudden cardiac death (SCD), aortic aneurysm, or other CV death. Non-CV death was defined as death caused by malignancy, infection/sepsis, respiratory, hemorrhage other than CV bleeding or stroke, renal, hepatobiliary or pancreatic, gastrointestinal, trauma, senility, or other non-CV death. Death from heart failure was defined as circulatory collapse related to hypotension or symptoms/signs of congestion at rest that may have required increased heart failure medication, including intravenous agents, in the days prior to onset. Patients may have had a previous history of heart failure or myocardial infarction. SCD was recorded in patients who 1) died suddenly and unexpectedly within 1 hour of cardiac symptom onset without preceding deterioration; 2) died unexpectedly during sleep; or, 3) died unexpectedly within 24 hours after last being seen alive. The diagnosis of SCD required the absence of severe pump failure death. Death due to ischemic stroke was defined according to the criteria for ischemic stroke. Acute onset of focal neurological deficit lasting more than 24 hours was defined as an ischemic stroke, and less than 24 hours was defined as a transient ischemic attack (TIA). Whether positive or negative, imaging data from computerized tomography (CT) or magnetic resonance imaging (MRI) of the brain were required to be uploaded into the web-based system. Major bleeding was defined according to the criteria published by the International Society of Thrombosis and Haemostasis (ISTH) [11].

### Statistical analysis

All statistical analyses were performed using SPSS Statistics software version 18.0 (SPSS, Inc., Chicago, IL, USA). Continuous normally distributed data are presented as mean plus/minus standard deviation (SD), and continuous non-normally distributed data are given as median and interquartile range (IQR). Categorical data are described as number and percentage. Comparisons of normally and non-normally distributed continuous data were performed using Student’s *t*-test and Mann-Whitney U test, respectively. For comparisons of categorical data, chi-square test or Fisher’s exact test was used. Hazard graphs were plotted to evaluate the increased risk of adverse outcome over time. A Cox proportional hazards model was used to compare factors that might influence the clinical outcome over time. Multivariate analysis was performed to identify factors that independently predict risk of outcome. The results of our multivariate analysis are shown as adjusted hazard ratio (aHR) and 95% confidence interval (95%CI). Data collection for this study was relatively complete with only left ventricular ejection fraction (LVEF) data missing in 15% of overall cases (512/3,405 patients), Imputation was employed to replace this missing data with substitute data. A *p*-value less than 0.05 was considered statistically significant for all tests and comparisons.

## Results

**Fig 1:**
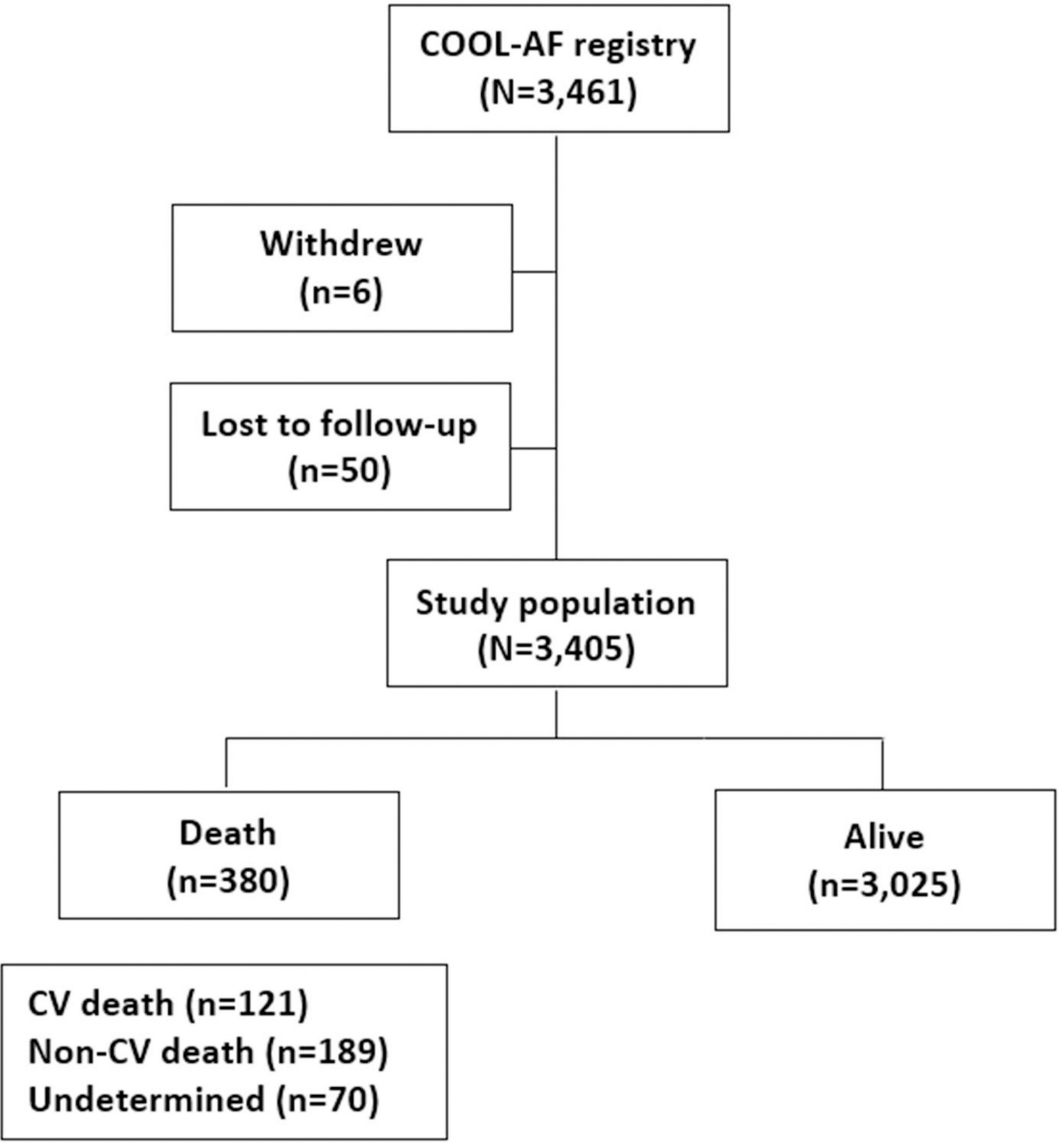
Flow diagram of patient enrollment and the flow of patients in this study. (Abbreviations: COOL-AF, COhort of antithrombotic use and Optimal INR Level in patients with non-valvular Atrial Fibrillation in Thailand; CV, cardiovascular).

We studied a total of 3,405 patients. The average age was 67.8±11.3 years, and 1,424 (41.8%) were female. Fig 1 shows a flow chart that describes the enrollment and flow of patients in this study.

### Outcomes

The mean follow-up duration was 31.8±8.7 months, and the median follow-up duration was 35.9 months (interquartile range [IQR]: 34.8–36.0) for a total follow-up duration of 9,026.7 person-years. Three hundred and eighty patients (11.2%) died during follow-up for an incidence rate of 4.25 per 100 person-years. Table 1 shows the baseline demographic and clinical characteristics of all patients, and compared between those who died and those who survived during follow-up. Patients who died had a significantly greater proportion of age greater than 70 years (68.4% *vs*. 41.7%, *p*<0.001), lower mean body mass index (23.6±4.6 *vs*. 25.4±4.7 kg/m^2^, *p*<0.001), lower mean diastolic blood pressure (72.9±13.7 *vs*. 75.5±12.5, *p*<0.001), higher proportion of permanent AF (55.5% *vs*. 46.3%, *p*<0.001), more comorbidities, and significantly greater use of warfarin (73.2% *vs*. 68.2%, *p* = 0.048).

**Table 1:** Baseline demographic and clinical characteristics of all patients, and compared between those who died and those who survived during follow-up.

Variables	All (N = 3,405)	Death (n = 380)	Survival (n = 3,025)	*p*-value
Age (years)	67.8±11.3	73.9±11.2	67.0±11.1	***<0*.*001***
Age ≥70 years	1,522 (44.7%)	260 (68.4%)	1,262 (41.7%)	***<0*.*001***
Female gender	1,424 (41.8%)	167 (43.9%)	1,257 (41.6%)	0.373
Body mass index (kg/m^2^)	25.2±4.7	23.6±4.6	25.4±4.7	***<0*.*001***
Systolic blood pressure (mmHg)	128.5±18.4	128.6±19.7	128.5±18.3	0.893
Diastolic blood pressure (mmHg)	75.2±12.7	72.9±13.7	75.5±12.5	***<0*.*001***
Pulse (bpm)	77.4±16.2	77.6±16.0	77.3±16.2	0.741
Time since diagnosis of AF (years)	3.4±4.3	3.5±4.7	3.4±4.3	0.519
Atrial fibrillation				***<0*.*001***
• Paroxysmal	1,148 (33.7%)	91 (23.9%)	1,057 (34.9%)	
• Persistent	645 (18.9%)	78 (20.5%)	567 (18.7%)	
• Permanent	1,612 (47.3%)	211 (55.5%)	1,401 (46.3%)	
Symptomatic AF	2,620 (76.9%)	286 (75.3%)	2,334 (77.2%)	0.409
History of heart failure	913 (26.8%)	139 (36.6%)	774 (25.6%)	***<0*.*001***
History of CAD	547 (16.1%)	89 (23.4%)	458 (15.1%)	***<0*.*001***
CIED	341 (10.0%)	47 (12.4%)	294 (9.7%)	0.105
History of ischemic stroke/TIA	592 (17.4%)	90 (23.7%)	502 (16.6%)	***0*.*001***
Diabetes mellitus	839 (24.6%)	126 (33.2%)	713 (23.6%)	***<0*.*001***
Hypertension	2,330 (68.4%)	286 (75.3%)	2,044 (67.6%)	***0*.*002***
Current smoker	678 (19.9%)	81 (21.3%)	597 (19.7%)	0.467
Dyslipidemia	1,917 (56.3%)	236 (62.1%)	1,681 (55.6%)	***0*.*015***
Renal replacement therapy	40 (1.2%)	12 (3.2%)	28 (0.9%)	***0*.*001***
Dementia	29 (0.9%)	8 (2.1%)	21 (0.7%)	***0*.*012***
Systemic embolism	25 (0.7%)	9 (2.4%)	16 (0.5%)	***0*.*001***
History of peripheral vascular disease	44 (1.3%)	12 (3.2%)	32 (1.1%)	***0*.*001***
History of carotid occlusive disease	8 (0.2%)	1 (0.3%)	7 (0.2%)	0.904
History of stent use	253 (46.2%)	44 (49.4%)	209 (45.5%)	0.499
History of CABG	65 (12.0%)	14 (15.7%)	51 (11.2%)	0.232
History of alcohol abuse	140 (4.1%)	14 (3.7%)	126 (4.2%)	0.656
History of bleeding	324 (9.5%)	54 (14.2%)	270 (8.9%)	***0*.*001***
CHA_2_DS_2_-VASc score	3.07±1.68	3.95±1.64	2.96±1.65	***<0*.*001***
• Low	287 (8.4%)	8 (2.1%)	279 (9.2%)	
• Intermediate	548 (16.1%)	24 (6.3%)	524 (17.3%)	
• High	2,570 (75.5%)	348 (91.6%)	2,222 (73.5%)	
HAS-BLED score	1.54±1.01	2.01±1.07	1.48±0.98	***<0*.*001***
• 0	490 (14.4%)	20 (5.3%)	470 (15.5%)	
• 1–2	2,375 (69.8%)	249 (65.5%)	2,126 (70.3%)	
• ≥3	540 (15.9%)	111 (29.2%)	429 (14.2%)	
Chronic kidney disease	1,756 (51.6%)	285 (75.0%)	1,471 (48.6%)	***<0*.*001***
Anemia	1,293 (38.0%)	228 (60.0%)	1,065 (35.5%)	***<0*.*001***
Left ventricular ejection fraction	60.2±13.7	59.1±15.0	60.3±13.6	0.129
Antiplatelet	892 (26.2%)	114 (30.0%)	778 (25.7%)	0.074
Anticoagulant	2,568 (75.4%)	296 (77.9%)	2,272 (75.1%)	0.234
• Warfarin	2,340 (68.7%)	278 (73.2%)	2,062 (68.2%)	***0*.*048***
• DOAC	228 (6.7%)	18 (4.7%)	210 (6.9%)	0.105
Beta blocker	2,477 (72.7%)	266 (70.0%)	2,211 (73.1%)	0.202
Calcium channel blocker	935 (27.5%)	130 (34.2%)	805 (26.6%)	***0*.*002***
Digitalis	539 (15.8%)	61 (16.1%)	478 (15.8%)	0.899
MRA	280 (8.2%)	43 (11.3%)	237 (7.8%)	***0*.*020***
Statin	2,014 (59.1%)	247 (65.0%)	1,767 (58.4%)	***0*.*014***
ACEI/ARB	1,557 (45.7%)	180 (47.4%)	1,377 (45.5%)	0.496
NSAID/Cox-2 inhibitor	83 (2.4%)	7 (1.8%)	76 (2.5%)	0.425

Data presented as mean ± standard deviation or number and percentage.

A *p*-value<0.05 indicates statistical significance (bold and italic).

**Abbreviations:** ACEI, angiotensin converting enzyme inhibitor; AF, atrial fibrillation; ARB, angiotensin receptor blocker; bpm, beats per minute; CABG, coronary artery bypass graft; CAD, coronary artery disease; CHA_2_DS_2_-VASc, congestive heart failure, hypertension, age ≥75 (doubled), diabetes, stroke (doubled), vascular disease, age 65 to 74 and sex category (female); CIED, cardiac implantable electronic device; DOAC, direct oral anticoagulant; HAS-BLED, Hypertension, Abnormal liver/renal function, Stroke history, Bleeding history or predisposition, Labile INR, Elderly, Drug/alcohol usage; MRA, mineralocorticoid receptor antagonist; NSAID, non-steroidal anti-inflammatory drug; TIA, transient ischemic attack.

### Causes of death

The causes and incidence rates of cardiovascular or non-cardiovascular death among the patients enrolled in this study are shown in Table 2. The incidence rate of CV death, non-CV death, and undetermined cause of death was 1.35, 2.11, and 0.78 per 100 person-years, respectively. CV death, non-CV death, and undetermined cause of death was found in 121 (31.8%), 189 (49.7%), and 70 (18.4%) patients, respectively. The rate of ischemic stroke death was 4.7%, and was nearly the same between patients taking and not taking OAC (4.7% *vs*. 4.8%, respectively). S2 Table shows the incidence rate per 100 person-years of all cause death, CV death, and non-CV death according to the pattern of OAC treatment. The adjusted hazard ratio specific to the effect of warfarin or DOAC (with or without TTR as a factor) compared to a comparator treatment condition on all cause death, CV death, and non-CV death is also shown. These data indicate that patients taking warfarin and having a TTR ≥65% had a lower rate of death compared with those taking warfarin and having a TTR <65%, and also when compared to patients not taking OAC.

**Table 2:** The causes of cardiovascular or non-cardiovascular death among the patients enrolled in this study.

Causes of death (N = 3,405)	n	% of the whole group (N = 3,405)	Rate per 100 person-years	% of those who died (n = 380)	% of those with a known cause of death (n = 310)	% of death
**Cardiovascular death**	**121**	3.6%	1.35	31.8%	39.0%	**% of CV death (n = 121)**
• Heart failure	31	0.9%	0.35	8.2%	10.0%	25.6%
• ICH	31	0.9%	0.35	8.2%	10.0%	25.6%
• Ischemic stroke	18	0.5%	0.20	4.7%	5.8%	14.9%
• SCD	21	0.6%	0.23	5.5%	6.8%	17.4%
• AMI	13	0.4%	0.15	3.4%	4.2%	10.7%
• Aortic aneurysm	2	0.1%	0.02	0.5%	0.6%	1.7%
• Other CV death	5	0.1%	0.06	1.3%	1.6%	4.1%
**Non-cardiovascular death**	**189**	5.6%	2.11	49.7%	61.0%	**% of non-CV death (n = 189)**
• Infection/sepsis	86	2.5%	0.96	22.6%	27.7%	45.5%
• Malignancy	17	0.5%	0.19	4.5%	5.5%	9.0%
• Hemorrhage other than CV bleeding or stroke	14	0.4%	0.16	3.7%	4.5%	7.4%
• Respiratory	16	0.5%	0.18	4.2%	5.2%	8.5%
• Senility	8	0.2%	0.09	2.1%	2.6%	4.2%
• Trauma	7	0.2%	0.08	1.8%	2.3%	3.7%
• Neurological	7	0.2%	0.08	1.8%	2.3%	3.7%
• Renal	5	0.1%	0.06	1.3%	1.6%	2.6%
• Hepatobiliary or pancreatic	4	0.1%	0.04	1.1%	1.3%	2.1%
• Gastrointestinal	2	0.1%	0.02	0.5%	0.6%	1.1%
• Other non-CV death	23	0.7%	0.26	6.1%	7.4%	12.2%
**Undetermined**	**70**	**2.1%**	0.78	**18.4%**		
**Total**	**380**	**11.2%**	4.25	**100.0%**		

**Abbreviations:** AMI, acute myocardial infarction; CV, cardiovascular; ICH, intracranial hemorrhage; SCD, sudden cardiac death.

Among those with a known cause of death, the most prevalent causes of CV death were heart failure (n = 31, 10%), intracranial hemorrhage (ICH; n = 31, 10%), sudden cardiac death (n = 21, 6.8%) ischemic stroke (n = 18, 5.8%), and acute myocardial infarction (n = 13, 4.2%). Concerning non-CV death, the most prevalent causes of death were infection/sepsis (n = 86, 27.7%), malignancy (n = 17, 5.5%), respiratory (n = 16, 5.2%), and hemorrhage other than CV bleeding or stroke (n = 14, 4.5%).

**Fig 2:**
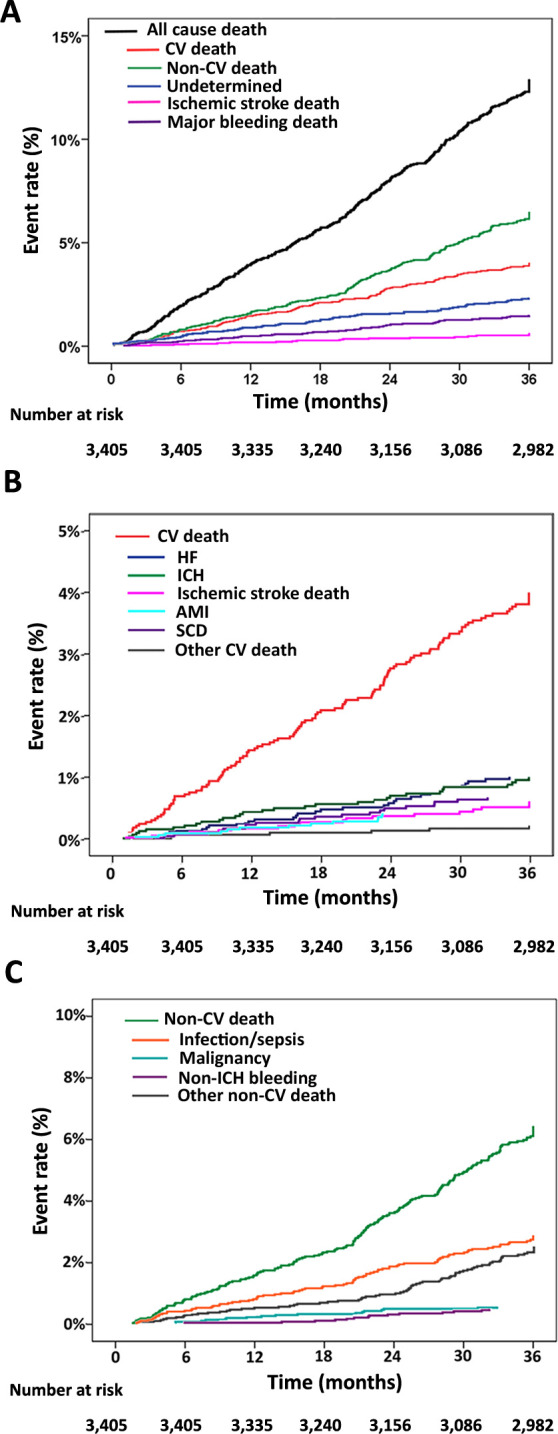
Cumulative event rate of (A) all death, CV death, non-CV death, undetermined death, ischemic stroke death, and major bleeding death; (B) the components of CV death; and, (C) the components of non-CV death. (**Abbreviations:** AMI, acute myocardial infarction; CV, cardiovascular; HF, heart failure; ICH, intracranial hemorrhage; SCD, sudden cardiac death).

Fig 2 shows the cumulative event rate of all death, CV death, non-CV death, undetermined cause of death, ischemic stroke death, and major bleeding death; the components of CV death; and, the components of non-CV death. The rate of non-CV death was higher than the rate of CV death throughout the follow-up period. The main causes of CV death were heart failure and ICH, and the rates of these two causes of death were at similar levels throughout the study period. Females demonstrated a trend toward an increased risk of death due to ischemic stroke compared to males. No significant difference between genders was observed for any of the other causes of death.

### Subgroup analyses

S1 Fig shows the incidence rates of clinical outcomes per 100 person-years compared between OAC and no OAC, warfarin with TTR <65% and ≥65%, and warfarin and DOAC. These data indicate that OAC increased the crude risk of ICH death more than two-fold compared to the no OAC groups. Warfarin with a TTR <65% increased the risk of ICH death more than two-fold when compared to warfarin with a TTR ≥65%. Similarly, warfarin increased risk of ICH death more than two-fold compared to DOACs.

Deaths related to bleeding, such as ICH and non-ICH major bleeding, tended to be increased in patients on OAC compared to no OAC, in those taking warfarin with a TTR <65% compared to ≥65%, and in those taking warfarin compared to DOACs.

S1 Table shows the incidence rate of fatal and all ischemic stroke, major bleeding, and ICH according to the pattern of OAC treatment. Among overall events, OAC decreased ischemic stroke from 1.93 to 1.37 per 100 person-years, but increased major bleeding from 1.13 to 2.63 per 100 person-years, and increased ICH from 0.27 to 0.95 per 100 person-years.

Despite a decrease in the ischemic stroke incidence rate, warfarin increased the rate of major bleeding and ICH. DOACs exerted greater benefit compared to warfarin relative to a reduction in ischemic stroke, major bleeding, and ICH. Regarding fatal events, OAC did not reduce fatal ischemic stroke, but did increase the incidence rate of major bleeding and ICH. The crude incidence rates of ischemic stroke, major bleeding and ICH among those taking DOACs were similar to those from patients not taking OAC, and were much lower than the incidence rates from those taking warfarin. Among those taking warfarin, patients with a TTR ≥65% had a lower incidence of ischemic stroke, major bleeding, and ICH compared to those with a TTR <65%.

**Fig 3:**
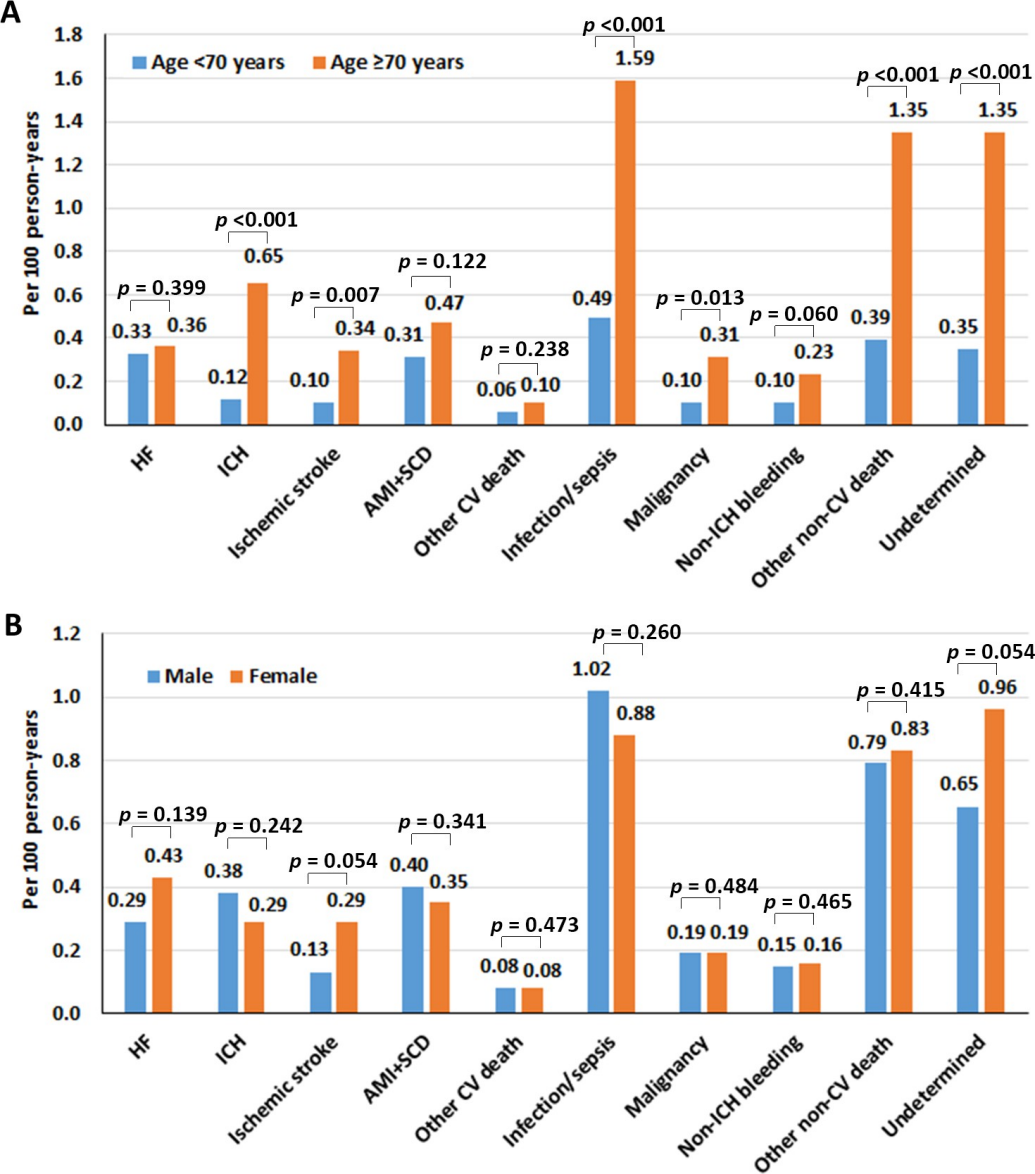
The causes of death per 100 person-years compared between patients aged <70 and ≥70 years (A), and compared between males and females (B). A *p*-value<0.05 indicates statistical significance. (**Abbreviations:** AMI, acute myocardial infarction; CV, cardiovascular; HF, heart failure; ICH, intracranial hemorrhage; SCD, sudden cardiac death).

Fig 3 shows the causes of death per 100 person-years compared between patients aged <70 and ≥70 years, and compared between males and females. Patients aged ≥70 years had a significantly increased risk of death due to ischemic stroke, ICH, infection/sepsis, malignancy, and other non-CV death compared to those aged <70 years.

### Multivariate analysis

**Fig 4:**
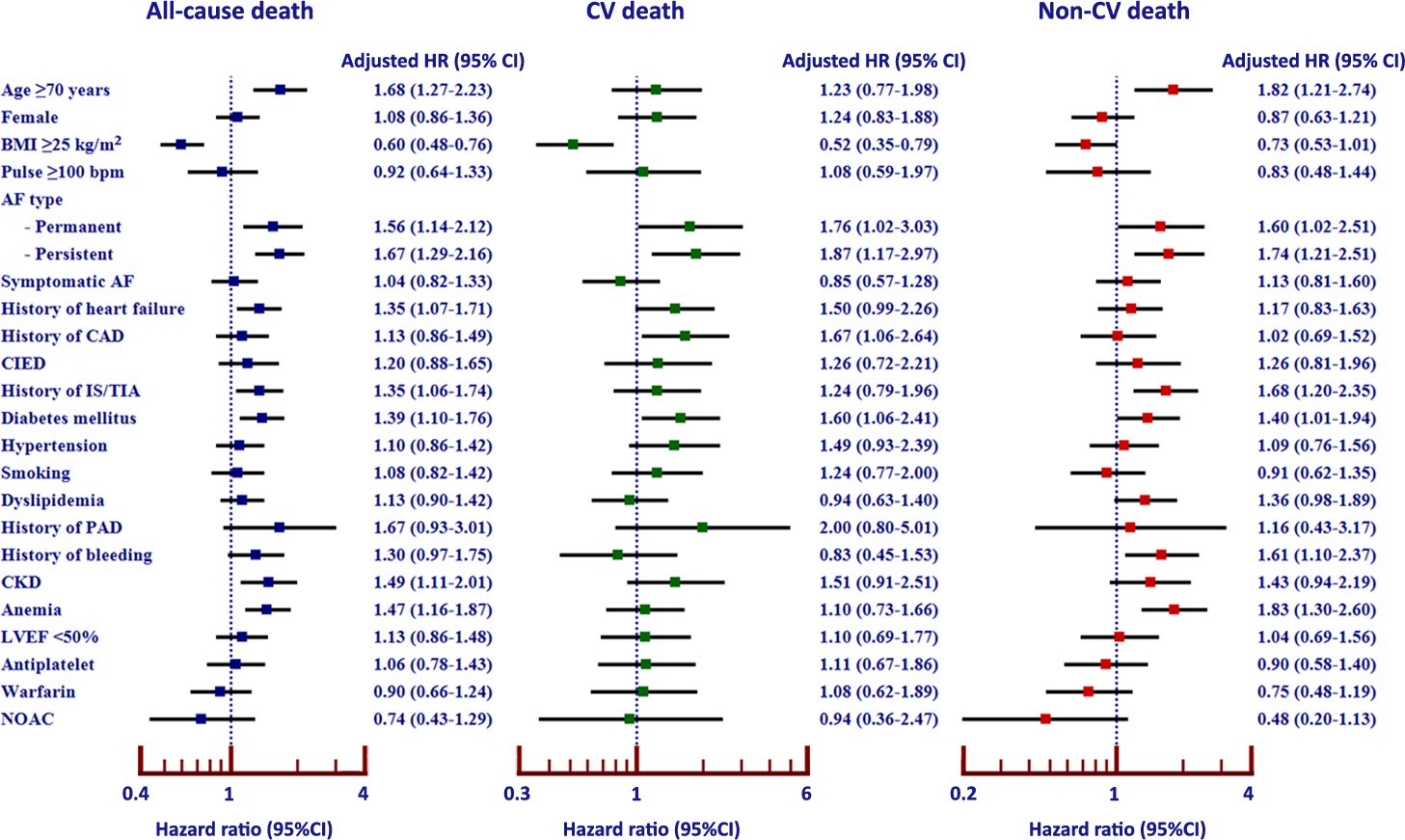
Forest plots showing the predictors of all-cause death, CV death, and non-CV death. (**Abbreviations:** AF, atrial fibrillation; AMI, acute myocardial infarction; BMI, body mass index; bpm, beats per minute; CAD, coronary artery disease; CI, confidence interval; CIED, cardiac implantable electronic device; CKD, chronic kidney disease; CV, cardiovascular; HF, heart failure; HR, hazard ratio; ICH, intracranial hemorrhage; IS, ischemic stroke; LVEF, left ventricular ejection fraction; NOAC, non-vitamin K antagonist oral anticoagulant; PAD, peripheral arterial disease; SCD, sudden cardiac death; TIA, transient ischemic attack).

The independent predictors of all-cause death from multivariate analysis were older age, low body mass index (BMI), persistent or permanent AF, history of heart failure, ischemic stroke, type 2 diabetes mellitus (T2D), CKD, and anemia (Fig 4). The independent predictors of CV death were low BMI, persistent or permanent AF, history of CAD, and T2D. The independent predictors of non-CV death were persistent or permanent AF, history of ischemic stroke, T2D, history of bleeding, and anemia (Fig 4).

## Discussion

The results of this prospective multicenter nationwide AF registry found that death due to ischemic stroke accounts for only 4.7% of all deaths in Asian patients with AF. This finding suggests that in addition to seeking to prevent ischemic stroke via the use of OACs, the management of Asian patients with AF should include a more holistic or integrated care approach to patient management in order to prevent other types of CV death and non-CV death.

Stroke prevention in patients with AF is a major goal of treatment many clinical practice guidelines [12–15]. Ischemic stroke from AF leads to more disability and higher mortality compared to non-AF-related stroke [16,17]. As such, one might perceive that to reduce mortality in patients with AF, we should focus only on the use of OAC; however, OAC use has both benefits and drawbacks. Although OAC does confer stroke prevention benefit, it is at the same time associated with an increased risk of bleeding, which is sometimes fatal [18]. Therefore, a careful evaluation of the risks and benefits of OAC is needed before starting OAC treatment, and shared decision-making between the physician and the patient/family is recommended [12].

Concerning the increased risk of bleeding in AF patients on OAC, ICH was reported to have a 30-day mortality rate of 43% compared to 9% for non-ICH bleeding [18]. Data from the Get With The Guidelines–Stroke registry during 2013–2016 revealed that among 141,311 patients with ICH, the use of DOACs was associated with significantly lower in-hospital mortality compared to warfarin (adjusted odds ratio: 0.75) [19].

Patients with AF commonly have multimorbidity, including T2D, hypertension, and renal dysfunction, which leads to death from many other causes besides ischemic stroke [20,21]. Management adherence to a holistic or integrated care pathway has been introduced to emphasize not only stroke prevention, but also patient-centered symptom-directed decisions on rate or rhythm control, and comorbidity and lifestyle management [22]. However, a multidisciplinary strategy is needed to provide comprehensive care to AF patients since this level of comprehensive care could not be managed or delivered by the cardiologist alone.

Table 3 summarizes the causes of death among patients from the COOL-AF study compared to those reported from 5 other previously published studies [20,21,23–25]. These studies include the GARFIELD-AF study [21], the FUSHIMI-AF study [25], the Loire Valley AF project [20], national insurance data [24], and DOAC clinical trials [23]. The incidence rate of all-cause death in AF patients was 3.8–6.3 per 100 person-years, CV death accounted for 26–64% of all deaths, and CV death accounted for 32–68% among those with a known cause of death. Most of the published data indicates that CV death accounts for less than half of all-cause deaths, while non-CV death accounted for more than half of all deaths. Among CV deaths, heart failure was the most common cause of CV death followed by SCD and AMI.

**Table 3:** Cause of death of patients with atrial fibrillation from COOL-AF study compared to some of the previously published data.

Cause of death (N = 3,405)	COOL-AF	GARFIELD [26]	Loire Valley-AF [20]	Korean-NHIS [24]	FUSHIMI [25]	4 DOAC trials [23]
Year of enrollment	2014–2017	2010–2014	2000–2010	2002–2013	2011–2012	2005–2010
Number of patients	3405	28628	8962	15411	4045	71683
Country	Thailand	Global	France, UK, Denmark	Korea	Japan	Global
Asian	100	25	NA	100	100	6.5–15.4
Age (Mean or median))	67.8	71	71	63.9	73.6	70–73
Female	41.8	44.4	38	40.2	40	35–40
Diabetes mellitus	24.6	21.7	15	22	23	23–40
Hypertension	68.4	77.5	41	64	63	79–94
Dyslipidemia	56.3	41	15	42.1	44	NA^a^
Smoking	19.9	34.8	12	18.5	NA	NA
Heart failure	26.8	20.6	49	24	27	32–63
CAD	16.1	19.9	20	34.8	15	14–17^b^
Ischemic stroke/TIA	17.4	12	8	15.5	18	19–55
CKD stage 3–5		10.4	7	NA	49	19–21
Permanent AF	47.3	12.7	38	10.3	41.1	67–83
CHA_2_DS_2_-VASc score (mean or median)	3.1	3	3.0	2.9	3.38	2.1–3.5^c^
HAS-BLED score (mean or median)	1.5	1.5	1.5	1.2	1.75	1.84
OAC	75.4	63.3	58	17.9	54	100
DOACs	6.7	17	0	NA	10	50
Warfarin	68.7	46.3	58	NA	44	50
Antiplatelet	26.2	27.9	35	16.6	28	34
**Incidence rate of death**						
**All death**	4.25	3.83	5.5	6.3	5.5	4.63
**Cardiovascular death**	1.35	1.55	2.97	NA	0.9	2.91
**Non-cardiovascular death**	2.11	1.37	2.37	NA	3.1	1.37
**Undetermined**	0.78	0.91	0.16	NA	1.5	0.41
**Cause of Death**						
**Cardiovascular death**	31.8%	40.5	54.1	38	25.5	64
Heart Failure	8.2%	10.8	29.2	3.2	14.4	14
Intracranial hemorrhage	8.2%	NA	4.1	NA	2.1	6
Ischemic stroke	4.7%	5.1	6.7	7.8	4.8	6
Sudden cardiac death	5.5%	7.5	3.3	NA	0.6	27
Acute myocardial infarction	3.4%	5.9	NA	5.0	1.3	3
Aortic aneurysm	0.5%	NA	0.8	NA	NA	NA
Other CV death	1.3%	11.1	7.3	NA	2.2	6
**Non-cardiovascular death**	49.7%	35.8	42.7	NA	54	30
Infection/sepsis	22.6%	6.7	17.6	NA	17.3	8
Malignancy	4.5%	10.3	11.8	23.4	23.1	10
Hemorrhage that neither CV bleeding or stroke	3.7%	4.8^d^	2.6	NA	NA	2
Respiratory	4.2%	8.0	3.2	8.4	4.1	3
Senile	2.1%	NA	NA	4.5	NA	NA
Trauma	1.8%	NA	NA	NA	NA	1
Neurological	1.8%	NA	2.7	NA	NA	NA
Renal	1.3%	NA	1.6	NA	3.2	NA
Hepatobiliary or pancreatic	1.1%	NA	NA	NA	NA	0.3
Gastrointestinal	0.5%	NA	NA	NA	NA	NA
Others non-CV death	6.1%	10.8	6.0	NA	6.2	5
**Undetermined**	18.4%	23.7	3.2	NA	20.4	6

^a^NA = not available.

^b^History of myocardial infarction^.^

^c^CHAD_S_ score.

^d^GARFIELD reported fatal major bleeding separate from CV or non-CV cause of death which included fatal ICH and fatal bleeding from non-ICH cause.

**Abbreviations:** AF, atrial fibrillation; CHA2DS2-VASc, congestive heart failure, hypertension, age ≥75 (doubled), diabetes, stroke (doubled), vascular disease, age 65 to 74 and sex category (female); CHADs, congestive heart failure, hypertension, age ≥75 years, diabetes mellitus, stroke [double weight]; COOL-AF, COhort of antithrombotic use and Optimal INR Level in patients with non-valvular Atrial Fibrillation in Thailand; CV, cardiovascular; GARFIELD, Global Anticoagulant Registry in the Field-Atrial Fibrillation; HAS-BLED, Hypertension, Abnormal liver/renal function, Stroke history, Bleeding history or predisposition, Labile INR, Elderly, Drug/alcohol usage; ICH, intracranial hemorrhage; TIA, transient ischemic attack.

From previous studies, ICH accounted for 2–6% of all deaths. In our study and in contrast, ICH accounted for 8% of all deaths and was the shared leading cause of CV death with HF death. In the present study, OAC increased the crude risk of ICH death more than two-fold compared to the no OAC groups. Warfarin users with a TTR <65% had a two-fold increased risk of ICH death compared to those with a TTR ≥65%. Similarly, warfarin increased the risk of ICH death more than two-fold compared to DOAC users. Hence, giving OAC (particularly, warfarin with TTR <65%) for stroke prevention increased the risk of major bleeding that exceeded the benefit of preventing ischemic stroke. Asian population was reported to be at greater risk for major bleeding and ICH compared to non-Asians [27,28]. Moreover, the results of a previously published meta-analysis of DOACs trials revealed DOACs to be more effective and safe in Asians than in non-Asians [29]; therefore, DOACs should be the preferred OAC in Asian population.

The rates of death related to ICH or major bleeding in our study were higher than those reported from previous studies (Table 3). This finding may be related to a higher use of warfarin and lower use of DOACs in our study compared to other studies. Regarding non-CV death, our results show that the proportion of deaths from non-CV causes, especially infection or sepsis, was higher than previous studies. As such, the strategy to reduce mortality in patients with AF should not focus primarily on stroke prevention, but should include adjunct strategies to minimize bleeding complications and to appropriately prevent and treat infection.

### Limitations

This study has some mentionable limitations. First, the results of this study were derived from data collected from large tertiary hospitals, which could limit their generalizability to community-based populations. Second, the cause of death was undetermined in 18.4% of patients who died, but this proportion is similar to those reported from previous studies, as shown in Table 3. Third, our study reports associations, but not causality. Fourth, the main OAC used in our study was warfarin (which is commonly used globally, especially in low-to-middle income countries) and the rate of DOAC use was comparatively low, which may limit the generalizability of our results. Fifth, his study results aren’t generalisable across the world and current trends of DOAC use. The results are important at local level especially in low-middle income countries. Efforts in prescribing DOACS instead of warfarin or in alternative, in obtaining better TTR should be encouraged in your population. Finally, we did not have data specific to the causes of death among healthy population to perform age- and gender-matched comparisons with the patients in our study cohort. This is due to the fact that the registry from which our data was collected enrolled only AF patients.

## Conclusion

Death due to ischemic stroke was responsible for only 4.7% of all deaths in Asian AF patients. Non-CV death, such as infection/sepsis or malignancy, was more far more prevalent than CV death in Asian AF patients. The use and type of OAC were found to be major determinants of ICH and major bleeding incidence. An improved integrated care approach is needed to reduce the prevalence of non-CV death in Asian AF patients.

## Supporting information

S1 Fig The incidence rates of clinical outcomes per 100 person-years compared between OAC and no OAC (A); warfarin with TTR <65% and ≥65% (B); and, warfarin and DOAC. A p-value<0.05 indicates statistical significance.
(PDF)

S1 Table Incidence rate of patient outcomes per 100 person-years according to the type of treatment and the time in therapeutic range. (PDF)

S2 Table Incidence rate per 100 person-years of all cause death, CV death, and non-CV death according to the pattern of OAC treatment. Adjusted hazard ratio specific to the effect of warfarin or DOAC (with or without TTR as a factor) compared to a comparator treatment condition on all cause death, CV death, and nonCV death.
(PDF)
