# Supplementary material for: Causes of death of patients with non-valvular atrial fibrillation in Asians
Source: PLoS One. 2023 Mar 1;18(3):e0282455. doi: 10.1371/journal.pone.0282455 (PMC9977019; doi:10.1371/journal.pone.0282455)
Supplement: S1 Fig — The incidence rates of clinical outcomes per 100 person-years compared between OAC and no OAC (A); warfarin with TTR <65% and ≥65% (B); and, warfarin and DOAC. A p-value<0.05 indicates statistical significance. (PDF) [file pone.0282455.s001.pdf]

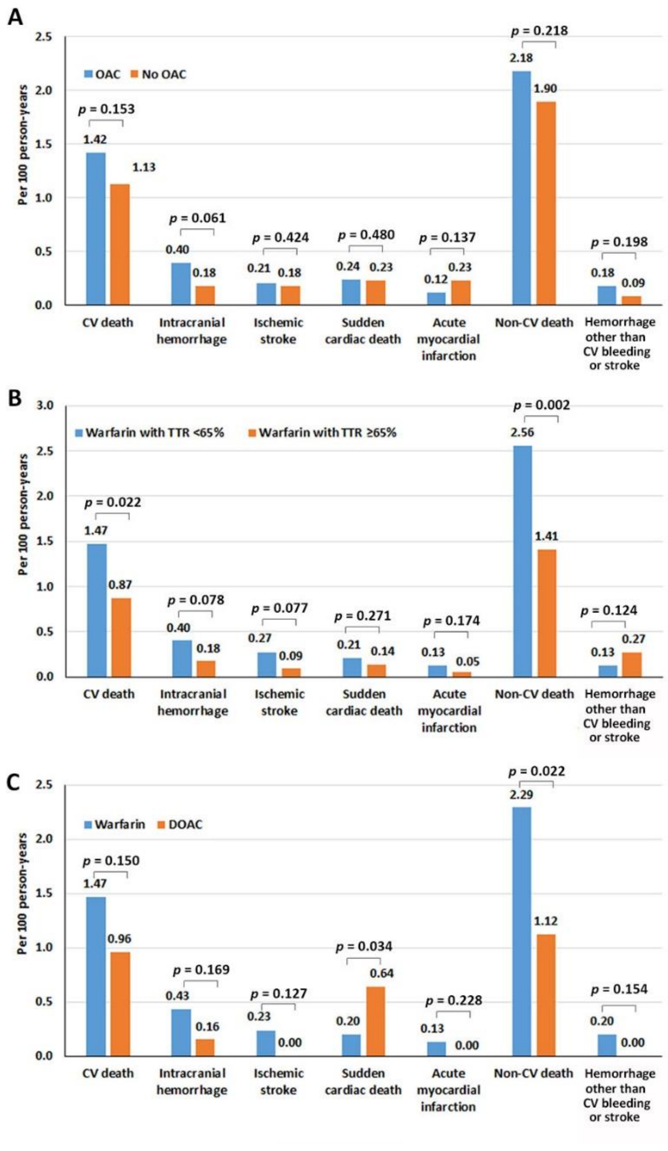

S1 Fig. The incidence rates of clinical outcomes per 100 person-years compared between OAC and no OAC (A); warfarin with TTR <65% and ≥65% (B); and, warfarin and DOAC. A *p*-value<0.05 indicates statistical significance.

(**Abbreviations:** CV, cardiovascular; DOAC, direct oral anticoagulant; OAC, oral anticoagulants; TTR, time in therapeutic range)
