# Supplementary material for: Causes of death of patients with non-valvular atrial fibrillation in Asians
Source: PLoS One. 2023 Mar 1;18(3):e0282455. doi: 10.1371/journal.pone.0282455 (PMC9977019; doi:10.1371/journal.pone.0282455)
Supplement: S1 Table — (PDF) [file pone.0282455.s002.pdf]

S1 Table. Incidence rate of patient outcomes per 100 person-years according to the type of treatment and the time in therapeutic range

Treatment / TTR	Fatal IS/SSE Rate (95%CI)	Fatal MB Rate (95%CI)	Fatal ICH Rate (95%CI)	All IS/SSE Rate (95%CI)	All MB Rate (95%CI)	All ICH Rate (95%CI)
All patients	0.20 (0.12-0.32)	0.5 (0.36-0.67)	0.34 (0.23-0.49)	1.51 (1.26-1.78)	2.25 (1.95-2.59)	0.78 (0.61-0.98)
No OAC	0.18 (0.05-0.46)	0.27 (0.10-0.59)	0.18 (0.05-0.46)	1.93 (1.39-2.60)	1.13 (0.73-1.67)	0.27 (0.10-0.59)
OAC	0.21 (0.11-0.35)	0.57 (0.41-0.78)	0.40 (0.26-0.58)	1.37 (1.10-1.68)	2.63 (2.25-3.04)	0.95 (0.73-1.21)
Warfarin	0.00 (0.00-0.00)	0.16 (0.01-0.88)	0.16 (0.01-0.88)	0.8 (0.26-1.87)	1.78 (0.89-3.18)	0.80 (0.26-1.86)
DOACs	0.23 (0.12-0.38)	0.62 (0.44-0.85)	0.42 (0.28-0.62)	1.43 (1.14-1.76)	2.72 (2.31-3.17)	0.96 (0.73-1.24)
TTR<65	0.26 (0.13-0.49)	0.53 (0.32-0.82)	0.40 (0.22-0.66)	1.72 (1.32-2.19)	3.15 (2.60-3.78)	1.15 (0.83-1.55)
TTR ≥65	0.09 (0.01-0.33)	0.45 (0.22-0.83)	0.18 (0.05-0.46)	0.82 (0.49-1.29)	1.79 (1.28-2.45)	0.41 (0.19-0.78)

Abbreviations: CI, confidence interval; DOAC, direct oral anticoagulants; ICH, intracranial hemorrhage; IS, ischemic stroke; MB, major bleeding; OAC, oral anticoagulant; SSE, systemic embolism; TTR, time in therapeutic range
