# Supplementary material for: Causes of death of patients with non-valvular atrial fibrillation in Asians
Source: PLoS One. 2023 Mar 1;18(3):e0282455. doi: 10.1371/journal.pone.0282455 (PMC9977019; doi:10.1371/journal.pone.0282455)
Supplement: S2 Table — Adjusted hazard ratio specific to the effect of warfarin or DOAC (with or without TTR as a factor) compared to a comparator treatment condition on all cause death, CV death, and nonCV death. (PDF) [file pone.0282455.s003.pdf]

S2 Table.. Incidence rate per 100 person-years of all cause death, CV death, and non-CV death according to the pattern of OAC treatment. Adjusted hazard ratio specific to the effect of warfarin or DOAC (with or without TTR as a factor) compared to a comparator treatment condition on all cause death, CV death, and non-CV death.

	Rate per 100 person-years				
	**No OAC**	**Warfarin**	**Warfarin with TTR <65%**	**Warfarin with TTR ≥65%**	**DOAC**
	**Rate (95%CI)**	**Rate (95%CI)**	**Rate (95%CI)**	**Rate (95%CI)**	**Rate (95%CI)**
All-cause death	4.39 (3.91-4.92)	4.55 (4.03-5.12)	4.75 (4.07-5.5)	2.74 (2.09-3.53)	2.88 (1.71-4.55)
CV death	1.13 (0.73-1.67)	1.47 (1.18-1.81)	1.47 (1.10-1.90)	0.87 (0.52-1.35)	0.96 (0.35-2.09)
Non-CV death	1.90 (1.37-2.57)	2.29 (1.93-2.70)	2.56 (2.07-3.13)	1.41 (0.96-2.01)	1.12 (0.45-2.31)

	Warfarin *vs.* no OAC**		Warfarin with TTR <65% *vs.* no OAC**		Warfarin with TTR ≥65% *vs.* no OAC**		DOAC *vs.* no OAC**		DOAC *vs.* warfarin**		Warfarin with TTR ≥65% vs. warfarin with TTR <65%**		DOAC *vs. w*arfarin with TTR ≥65%**		DOAC *vs.* warfarin with TTR <65%**	
	aHR* (95%CI)	*p*	aHR* (95%CI)	*p*	aHR* (95%CI)	*p*	aHR* (95%CI)	*p*	aHR* (95%CI)	*p*	aHR* (95%CI)	*p*	aHR* (95%CI)	*p*	aHR* (95%CI)	*p*
All-cause death	0.91 (0.71-1.18)	0.476	0.97 (0.76-1.24)	0.808	0.54 (0.38-0.77)	***0.001***	0.75 (0.45-1.27)	0.288	0.81 (0.50-1.33)	0.415	0.62 (0.46-0.83)	***0.002***	1.24 (0.71-2.14)	0.451	0.83 (0.50-1.36)	0.456
CV death	1.07 (0.67-1.70)	0.775	1.12 (0.73-1.72)	0.610	0.66 (0.34-1.27)	0.211	1.28 (0.50-3.29)	0.602	0.88 (0.38-2.03)	0.757	0.67 (0.39-1.13)	0.131	1.44 (0.55-3.80)	0.463	0.87 (0.37-2.07)	0.754
Non-CV death	0.85 (0.59-1.22)	0.375	0.78 (0.54-1.12)	0.183	0.51 (0.31-0.84)	***0.008***	0.50 (0.22-1.13)	0.094	0.64 (0.30-1.38)	0.252	0.59 (0.39-0.89)	***0.011***	1.01 (0.43-2.39)	0.982	0.61 (0.28-1.34)	0.222

A *p*-value<0.05 indicates statistical significance

* Adjusted hazard ratio was adjusted for age ≥70 years, female gender, body mass index (kg/m^2), type of atrial fibrillation, history of heart failure, history of coronary artery disease, history of ischemic stroke/transient ischemic attack, diabetes mellitus, hypertension, current smoker status, dyslipidemia, renal replacement therapy, history of peripheral vascular disease, history of stent use, history of coronary artery bypass graft, and history of bleeding

** Comparator group

Abbreviations: aHR, adjusted hazard ratio; CI, confidence interval; CV, cardiovascular; DOAC, direct oral anticoagulant; OAC, oral anticoagulant; TTR, time in therapeutic range
